# Diverging gaps in childcare time by parental education in South Korea

**DOI:** 10.4054/demres.2021.44.6

**Published:** 2021-01-26

**Authors:** Hyunjoon Park

**Affiliations:** 1 Department of Sociology, University of Pennsylvania, USA.

## Abstract

**BACKGROUND:**

Parental time is a key resource for children’s development. Studies in the United States highlight diverging gaps in parental time for children between highly educated and low-educated parents. South Korea offers an interesting context in which to examine the trend.

**OBJECTIVE:**

This study assesses whether differences in childcare time have diverged or converged between parents with higher and lower levels of education over the 15-year period. Utilizing the advantage of household survey, the total amount of childcare time spent by both fathers and mothers is examined, in addition to separate time for each parent.

**METHODS:**

The Korean Time Use Surveys (KTUS), conducted in 1999, 2004, 2009, and 2014, provide time diary data for two consecutive days. OLS regression models are applied to 14,044 married mothers and fathers who have at least one child under school age in order to examine how educational differences in childcare time have changed across the four surveys.

**RESULTS:**

The OLS results show that both mothers and fathers have spent increasingly more time for childcare between 1999 and 2014, regardless of educational levels. However, the rise of time use is more substantial among mothers and fathers with a university degree than their counterparts with high school or less education. The diverging trend is even more evident for the combined childcare time spent by both mothers and fathers.

**CONTRIBUTION:**

The divergence in childcare time by parental education is consistent with emerging trends of growing educational gaps in family behavior in Korea, raising the concern for diverging destinies between advantaged and disadvantaged children.

## 1. Introduction

The extensive involvement of parents, particularly mothers, in children’s education in South Korea (Korea, hereafter) is well known (Park and Abelmann 2004; Park, Byun, and Kim 2011). Shifting norms for parenting increasingly request ‘involved fathers,’ emphasizing the roles of fathers as well as mothers for children’s well-being. Studies show that both Korean fathers and mothers have increasingly spent more time for childcare, and also that parental education tends to be positively associated with childcare time (Lee, Park, and Sanida 2020; Song 2011). However, it is not clear whether such increase in parents’ childcare time is uniform across parents of varying socioeconomic status. Studies provide evidence of growing gaps in childcare time between more- and less-educated parents in the United States and some European countries, supporting the argument of diverging destinies (Altintas 2016; Dotti Sani and Treas 2016; McLanahan 2004).

Some distinctive features of Korean society make it difficult to expect divergence in childcare time by parental education. The high level of parental involvement regardless of social class, a tenuous relationship between mother’s education and paid work, and father’s long hours of working suggest limited differentiation in childcare time by parental education (Brinton 2001; OECD 2019). However, studies of family behavior in Korea reveal growing divergence in the likelihood of marriage and divorce between the most- and least-educated men and women, especially under the context of rising economic inequality (Park and Lee 2017; Park and Raymo 2013). This evidence of diverging family behavior suggests a similar trend in childcare time by parental education.

Drawing on time diary data collected in Korea between years 1999 and 2014, I assess whether differences in childcare time for children under school age between more-and less-educated parents diverged over time. The trends are examined for mothers and fathers, separately, and also for combined time of both parents. The Korean case offers an opportunity of assessing generability of diverging destinies with respect to parents’ childcare time, which is a critical resource for children’s education and well-being. The converging trend may not only challenge the general pattern of diverging destinies but it may also indicate the role of parenting in ameliorating educational inequality. But if diverging, the trend would add another challenge to policy efforts to reduce educational inequality.

## 2. Data and methods

The Korean Time Use Survey (KTUS) is a cross-sectional survey of nationally representative households and their members, conducted every five years since 1999. All members of selected households, aged 10 and older, recorded their primary and secondary (simultaneous) activities in the time diary in 10-minute units for the entire 24 hours for two consecutive days. The current study includes only households with at least one child under school age given the focus on parental time to take care of children before entering school. Only mothers and fathers, who are both aged 18 to 49, currently married and live in the same household as the household head or the head’s spouse, are included.

The 1999, 2004, and 2009 KTUS datasets contain minutes for childcare activities specifically for children under school age. However, in 2014 data pertain to childcare activities for children under age 10, not school age. Therefore, childcare time in 2014 may not refer to time only for children under school age if there is at least one additional child below age 10 but attending school. Studies show that parental educational differences in time and type of parental involvement depend on the age, and probably number, of children (Gracia 2014; Kalil, Ryan, and Corey 2012).

If the number of children attending school but under age 10 (proximately ages 7–9) was known in 1999, 2004, and 2009 KTUS, I would include only parents with at least one child under school age and no other children between ages 7 and 9 across all four – surveys. However, the number of children aged 7 to 9 is known only in 2014. Eventually, I decided to restrict the sample for 2014 to parents who have at least one child under school age and no other children between ages 7 and 9. This decision was to guarantee childcare time for ‘children under age 10’ to be the same as time for ‘children under school age’ in 2014.

In sum, the 2014 sample excludes parents who have at least one child under school age but also another children between ages 7 and 9, while the samples for 1999, 2004, and 2009 consist of parents who have at least one child under school age but for whom the number of additional children aged 7 to 9 is not known. To check robustness of the result, I used the original sample for 2014 (all parents who have at least one child under school age regardless of whether having another child between ages 7 and 9), assuming childcare time for children under age 10 in 2014 to be childcare time for children under school age. The results with the whole sample were hardly different from those based on the restricted sample. For another robust check, I selected parents with two or three children under school age (and thus likely have no additional child between ages 7 and 9) in 2009 and 2014 for which the total number of children under school age was available. The trend between 2009 and 2014 from this comparison was similar to that on the basis of the restricted sample for 2014.

The key outcome measure is childcare time spent daily by mothers and fathers as primary activities. Childcare includes all activities related to physical care, developmental care (reading to, helping homework, and playing with), health care, and other care-related activities. Childcare as a secondary activity, done simultaneously with a primary activity, is not considered. Each of mother’s and father’s education is distinguished into three categories, based on her or his highest level of education: (1) high school or less; (2) some college; (3) university (a bachelor’s degree or higher). Several demographic and socioeconomic variables are controlled. Table 1 presents unweighted descriptive statistics for all the variables used.

The following linear regression model is estimated to predict childcare time of mothers and fathers, separately:

(1) $${\text{Minutes }}_{ij}=a+b_{1}SC+b_{2}\text{Univ }+b_{3}Yr2004+b_{4}Yr2009+b_{5}Yr2014+b_{6}(SC_{-}Yr2004)\;+b_{7}(SC_{-}Yr2009)+b_{8}(SC_{-}Yr2014)+b_{9}({\text{Univ }}_{-}Yr2004)\;+b_{10}({\text{Univ}}_{-}Yr2009)+b_{11}({\text{Univ }}_{-}Yr2014)+\sum_{12}^{24}b_{k}X_{k}$$

Each mother (or father) *i* has two observations for two days (*j*=1 or 2). The coefficient *b*_1_(*b*_2_) indicates the difference in childcare time between parents with some college (*SC*) (university – *Univ*) and parents with high school or less education. The interaction coefficients between some college and year, *b*_6_ through *b*_8_, indicate how larger or smaller the childcare time gap is between mothers with the lowest level of education in 2004, 2009, or 2014, respectively, than the corresponding gap in 1999. The trend in the gap between mothers with university education and mothers with the lowest level of education is presented by interaction coefficients between university and year, *b*_9_ through *b*_11_ Assuming that education positively associated with childcare time, increasingly positive coefficients of interaction across 2004, 2009, and 2014 indicate diverging trends in educational differences. Contrastingly, increasingly negative coefficients of interaction indicate convergence. No weights are used for regression analyses.

In addition to separate analyses for mothers and fathers, combined childcare time of both mothers and fathers is examined at the household level. A new measure of education at the household level is used. Couples are distinguished into three groups: (1) low-educated couples – both mothers and fathers have high school or less education; (2) middle-educated couples – mothers and fathers have different levels of education; and (3) high-educated couples – both mothers and fathers have a university degree. All control variables used in Equation (1) are included in this regression model at the household level, except for the number of time diary for the weekends (Saturday and Sunday) in replace of days of the time diary.

## 3. Results

In Table 2 for the linear regression analysis of childcare time, both gross and net educational differences are presented for mothers and fathers, respectively. Childcare time differences between mothers with high school or less education and mothers with university education continuously diverged across four years. In the reference year (i.e., 1999), mothers with university education spent 15 minutes more for childcare than mothers with high school or less education, after all other variables held constant. The interaction coefficients between university and each year show that the net difference increased to 23 (15 + 8), 31 (15 + 16), and 40 minutes (15 + 25) in 2004, 2009, and 2014, respectively. The coefficients of survey years indicate that for mothers with the lowest level of education, daily childcare time increased every survey from the baseline year.

The diverging gap in childcare time between the most- and least-educated mothers is similarly observed for gross differences (day of time diary controlled for). Among others, changes in mother’s age and work compensated each other to make the gross and net educational differences be similar. The mean age of the least-educated mothers increased across years more substantially than that of the most-educated mothers, while the share of working mothers slightly decreased among the least-educated but increased among the most-educated. Given the negative associations of childcare time with both mother’s age and work, controlling for mother’s age would make the diverging trend less substantial but controlling for mother’s work would do the opposite.

Figure 1a displays predicted daily childcare minutes for mothers with the lowest and highest levels of education, calculated from the coefficients presented in Table 2 with fixing all other controls at their means (the line for mothers with some college is not presented). The figure includes the point estimates of childcare minutes with 95% confidence intervals. The two lines for the most- and least-educated mothers diverged across years. In 1999, mothers with high school or less education were predicted to spend 125 minutes per day to care for their children under school age, 15 minutes less than their most-educated counterparts. Both groups increased childcare time across years. But the increase was more substantial among the most-educated mothers, leading to divergence. In 2014, childcare time reached 230 minutes per day among the most-educated mothers, 40 minutes more than the least-educated mothers. The 95% confidence intervals are not overlapped between the two groups of mothers

Turning to the result for fathers in Table 2, the patterns of interaction coefficients between educational levels and years are similar with those for mothers. The gross and net differences and their trends are very similar. Figure 1b presents predicted childcare minutes for fathers, calculated from the coefficients presented in Table 2. Only two lines for fathers with the lowest and highest levels of education are presented. Fathers spent less time for childcare than mothers. In 1999, the least-educated fathers spent only 22 minutes daily for childcare, 103 minutes less than mothers with the same level of education. The most-educated fathers spent 24 minutes per day, 117 minutes less than university-educated mothers. The negligible educational gap in fathers’ childcare time in 1999 increased to 6 and 14 minutes more by the most-educated fathers in 2004 and 2009, respectively. The gap slightly decreased to 12 minutes in 2014. Similar to mothers, the trend for fathers is consistent with diverging destinies.

The result in Table 3 is for combined childcare time of both mothers and fathers within the same households. The interaction coefficients between highly educated couples (both mothers and fathers have university education) and survey years increase continuously across years in both gross and net models. The sizes of interaction coefficients are larger than corresponding coefficients from separate models for mothers and fathers in Table 2. Moreover, the interaction terms between some college and survey years also show a similar pattern of rising educational gaps, which was not observed in separate analyses for mothers and fathers.

**Figure 2:**
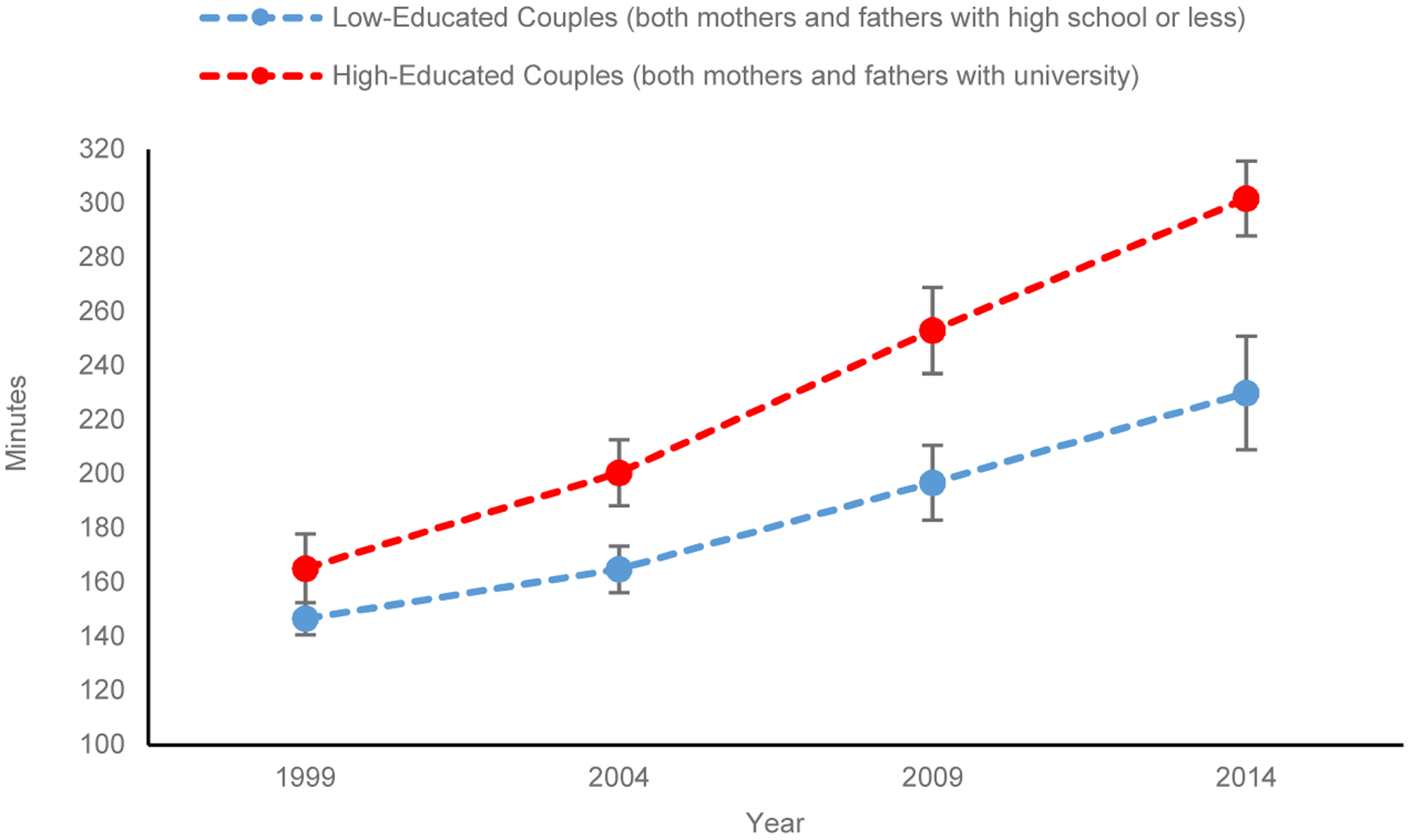
Predicted combined childcare minutes spent daily by both mothers and fathers (household level) (95% confidence intervals)

Figure 2 visualizes the diverging trend in combined childcare time of both mothers and fathers, based on the coefficients of the net model in Table 3 with all other variables fixed at their means. Presented are only two lines for households with both mothers and fathers with a university degree and their counterparts with both mothers and fathers having high school or less education. The predicted combined childcare time increased for both types of households. However, households with both mothers and fathers with university education displayed a more substantial increase across years than households with both mothers and fathers having high school or less education. In 1999, the former spent 165 minutes daily for childcare, 19 minutes more than the latter. The gap increased to 36 minutes in 2004, 56 minutes in 2009, and further to 72 minutes in 2014. No overlapped confidence intervals highlight significant differences in the estimated minutes between the two types of households.

## 4. Conclusion

From 1999 to 2014, Korean parents increasingly spent more time for childcare for both genders and across all levels of education. However, the increase was not uniform across parents with different levels of education, leading to divergence between those at the top and bottom of educational hierarchy. The diverging trend was consistent regardless of whether separate childcare time of mothers and fathers or combined time of both parents were examined. Notably, although the increase in the educational gap in childcare time between 1999 and 2014 was smaller for fathers than mothers in the absolute term, the increase in the relative term was larger for fathers than mothers. The diverging gap in childcare time for fathers as well as mothers is consistent with the argument of diverging destines that McLanahan (2004) highlighted as a key feature of the second demographic transition.

What might explain the diverging gap in childcare time by parental education in Korea? Rapid educational expansion reduced the share of parents with high school or less education across years, making the least-educated group more selective. Earlier I described how changes in the mean age and the share of working mothers contributed to the trend in the childcare time gap by parental education. Interestingly, the diverging trend between the most- and least-educated parents remained intact after controlling for compositional differences across parents with different levels of education. The remaining gaps in childcare time may reflect differences between the most- and least-educated parents in cultural orientations toward parenting norms, particularly intensive parenting (Dotti Sani and Treas 2016; Weininger, Lareau, and Conley 2015). However, it will be also useful for future research to assess how changes in not only observed but also unobserved characteristics contribute to the diverging trend by parental education.

Educational expansion could also alter the value and meaning of university education over time. I used the same educational categories across years despite changing educational distributions. Future research may utilize measures of relative education that consider relative ranking of people with a specific educational category (Shavit and Park 2016). Moreover, as previous studies in both Korea and other countries demonstrated, differences in childcare time by parental education may also depend on the age and type of childcare (Gracia 2014; Kalil, Ryan, and Corey 2012; Lee, Park, and Sanida 2020; Song 2011). Future studies may expand the current scope by comparing parental time for older age groups and for different care activities.

## Figures and Tables

**Figure 1a:**
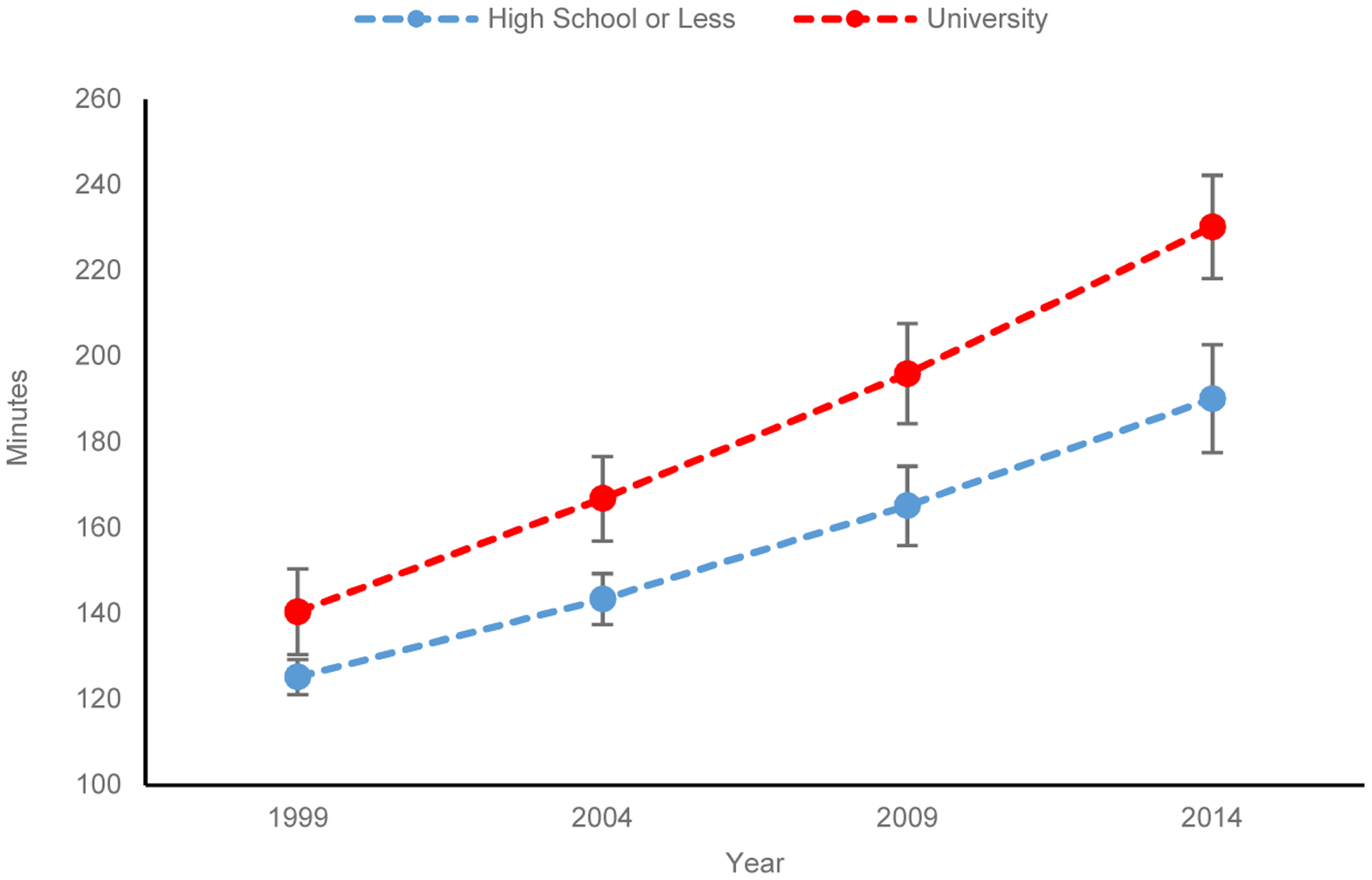
Predicted childcare minutes mothers spend daily (95% confidence intervals)

**Figure 1b:**
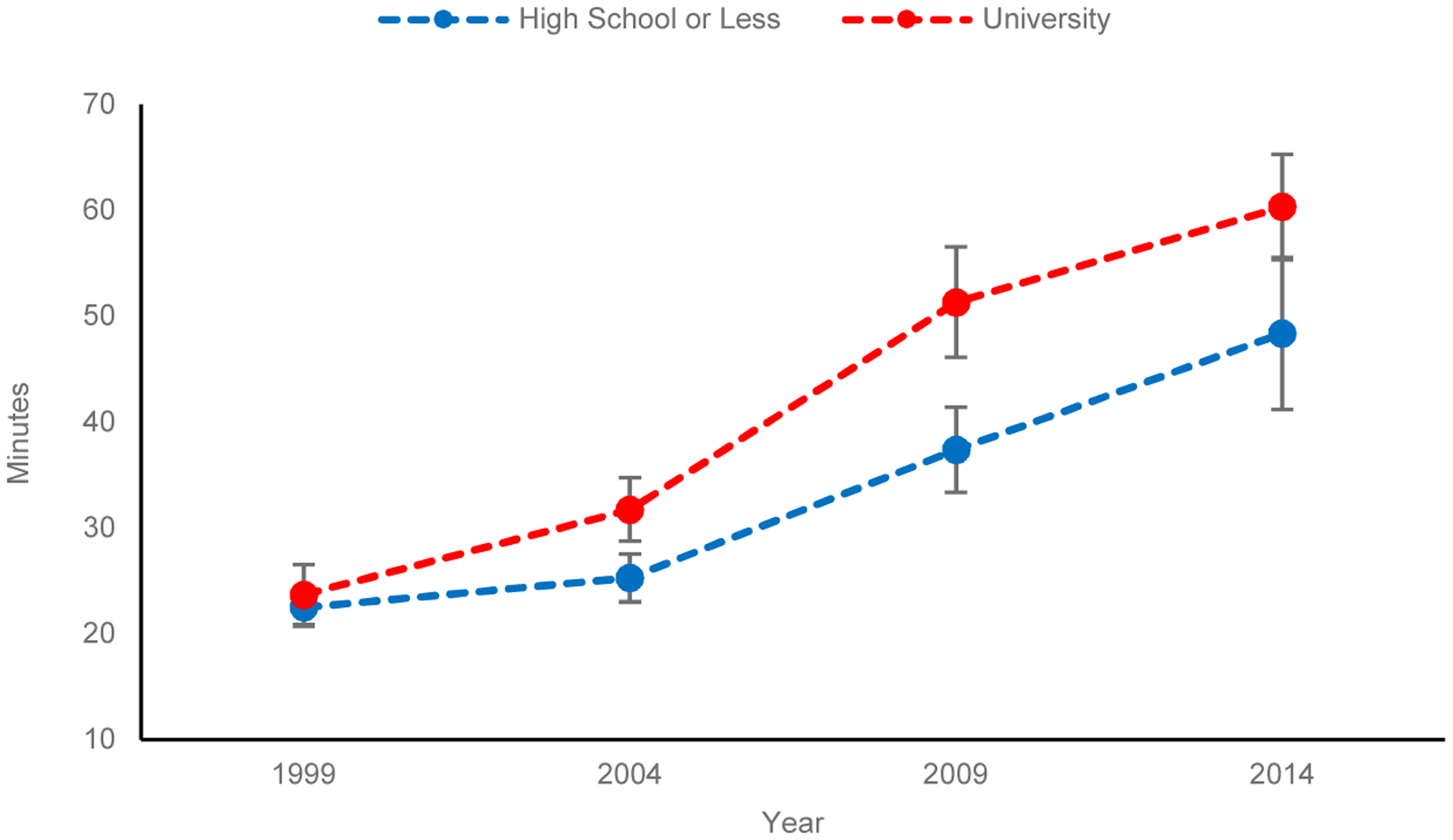
Predicted childcare minutes fathers spend daily (95% confidence intervals)

**Table 1:** Descriptive statistics of variables

	Mothers				Fathers			
	1999	2004	2009	2014	1999	2004	2009	2014
Education (%)								
High School or less	75.0	57.4	43.0	22.8	59.5	45.8	35.9	20.9
Some college	11.5	19.6	29.9	39.5	14.7	17.2	28.9	32.9
University	13.6	22.9	27.2	37.7	25.8	37.0	35.2	46.3
Working (%)	37.9	38.3	38.2	39.6				
Working hours (%)								
0–44					20.9	27.1	29.8	30.9
45–60					46.2	51.5	49.5	53.3
61 or more					32.9	21.4	20.8	15.8
Farm household (%)	3.4	2.4	1.5	1.1	3.4	2.4	1.5	1.1
Owning home (%)	43.7	48.5	53.7	55.5	43.7	48.5	53.7	55.5
Age^a^	31.5 (4.33)	32.5 (4.12)	33.4 (4.29)	33.9 (4.35)	34.7 (4.43)	35.3 (4.34)	35.9 (4.60)	36.1 (4.56)
Total number of children aged 10 to 18^a^	0.16 (0.46)	0.17 (0.48)	0.17 (0.49)	0.13 (0.41)	0.16 (0.46)	0.17 (0.48)	0.17 (0.49)	0.13 (0.41)
Day of time diary (%)								
Monday	10.3	9.8	9.6	9.9	10.3	9.8	9.6	9.9
Tuesday	10.1	9.7	8.6	10.7	10.1	9.7	8.6	10.7
Wednesday	10.0	9.7	8.6	10.7	10.0	9.7	8.6	10.7
Thursday	10.0	10.0	9.9	10.3	10.0	10.0	9.9	10.3
Friday	20.3	20.6	22.1	19.8	20.3	20.5	22.1	19.8
Saturday	19.8	20.4	21.9	19.2	19.8	20.4	21.9	19.2
Sunday	19.6	19.7	19.3	19.6	19.5	19.8	19.3	19.6

*Note*: all statistics are unweighted.

a For these variables, means and standard deviations in parentheses are presented.

N = 7,022 mothers with 14,044 observations; 7,022 fathers with 14,044 observations.

**Table 2:** Linear regression models of childcare time (minutes) for mothers and fathers, separately

	Mothers				Fathers			
		95% Conf.		95% Conf.		95% Conf.		95% Conf.
	b	Interval	b	Interval	b	Interval	b	Interval
Education (ref: High School or Less)								
Some College	21.526	[6.79, 36.26]	14.560	[1.66, 27.46]	4.241	[−0.11, 8.59]	3.388	[−0.84, 7.62]
University	12.594	[0.28, 24.91]	15.248	[4.45, 26.04]	1.579	[−1.83, 4.99]	1.216	[−2.12, 4.56]
Year (ref: 1999)	12.316	[4.11, 20.53]	18.223	[10.99, 25.46]	2.417	[−0.48, 5.32]	2.785	[−0.08, 5.65]
2009	27.107	[16.01, 38.20]	39.939	[29.86, 50.02]	13.454	[9.00, 17.91]	14.896	[10.52, 19.27]
2014	47.567	[32.21, 62.93]	64.925	[51.68, 78.17]	24.487	[16.89, 32.08]	25.880	[18.51, 33.26]
Education × Year								
Some College × 2004	4.831	[−15.55, 25.22]	4.430	[−13.37, 22.23]	4.374	[−2.23, 10.98]	2.608	[−3.84, 9.05]
Some College × 2009	−.5.574	[−27.68, 16.53]	−4.923	[−24.40, 14.55]	2.279	[−5.84, 10.40]	0.030	[−7.97, 8.03]
Some College × 2014	11.909	[−12.05, 35.87]	9.503	[−11.30, 30.31]	12.306	[1.68, 22.93]	10.207	[−0.21, 20.63]
University × 2004	−0.233	[−18.22, 17.76]	8.128	[−7.56, 23.81]	6.707	[1.60, 11.81]	5.259	[0.25, 10.27]
University × 2009	10.130	[−10.88, 31.14]	15.577	[−2.75, 33.91]	14.556	[7.01, 22.11]	12.720	[5.36, 20.08]
University × 2014	20.098	[−3.13, 43.32]	24.867	[4.55, 45.18]	12.978	[3.37, 22.59]	10.747	[1.36, 20.14]
Age (centered at 33/35)			−5.454	[−6.09, −4.82]			−0.871	[−1.13,−0.61]
Wife working (vs. not working)			−90.151	[−94.86, −85.44]			−0.874	[−3.01, 1.26]
Husband weekly working hours (0–44)								
45–60			5.260	[−0.63, 11.15]			−11.129	[−13.96, −8.30]
61 or more			12.662	[5.85, 19.47]			−17.846	[−20.85, −14.84]
Farm household (vs. not)			24.187	[−2.75, 33.91]			6.813	[−0.26, 13.89]
Owning home (vs. not)			−9.038	[−13.98,−4.09]			−4.479	[−6.58, −2.38]
Number of children aged 10 to 18			−23.929	[−28.80, −19.06]			−8.798	[−10.74, −6.86]
Day of Time Diary (ref: Monday)								
Tuesday	12.303	[2.71, 21.89]	14.879	[6.51, 23.24]	0.682	[−2.36, 3.72]	0.494	[−2.49, 3.48]
Wednesday	4.996	[−4.45, 14.45]	7.581	[−0.71, 15.87]	2.649	[−0.59, 5.89]	2.407	[−0.77, 5.58]
Thursday	0.748	[−8.48, 9.98]	3.374	[−4.67, 11.42]	0.712	[−2.32, 3.75]	0.693	[−2.29, 3.68]
Friday	5.847	[−2.12, 13.81]	6.467	[−0.50, 13.44]	1.542	[−1.12, 4.21]	1.461	[−1.17, 4.09]
Saturday	−6.063	[−14.00, 1.88]	−6.415	[−13.47, 0.64]	15.612	[12.52, 18.70]	15.227	[12.19, 18.27]
Sunday	−23.773	[−29.59, −17.96]	−23.503	[−28.90, −18.10]	28.597	[25.41, 31.78]	28.247	[25.07, 31.42]
Constant	134.368	[126.97, 141.76]	160.491	[151.76, 169.22]	12.581	[9.88, 15.29]	27.193	[23.44, 30.94]
R^2^	0.048		0.240		0.095		0.123	
N	14,044 (for 7,022 mothers)				14,044 (for 7,022 fathers)			

*Note*: 95% confidence intervals are based on robust clustered standard errors.

**Table 3:** Linear regression models of combined childcare time (minutes) by both mothers and fathers (household level)

	b	95% Conf. Interval	b	95% Conf. Interval
Couple’s education (ref: Low - both mothers and fathers with high school or less)				
Middle (mothers and fathers with different levels of education)	18.728	[7.95, 29.51]	9.198	[−0.47, 18.87]
High (both mothers and fathers with university)	17.132	[1.50, 32.76]	18.577	[4.55, 32.61]
Year (ref: 1999)				
2004	13.803	[2.20, 25.41]	18.246	[7.86, 28.63]
2009	36.513	[19.82, 53.21]	50.185	[35.20, 65.17]
2014	61.712	[37.40, 86.02]	83.370	[61.56, 105.18]
Couple’s education × Year				
Middle × 2004	8.857	[−8.44, 26.15]	13.688	[−1.75, 29.12]
Middle × 2009	10.609	[−11.25, 32.47]	13.228	[−6.28, 32.74]
Middle × 2014	34.490	[6.67, 62.31]	30.649	[5.82, 55.48]
High × 2004	9.120	[−13.72, 31.96]	17.113	[−3.28, 37.51]
High × 2009	31.861	[3.68, 60.04]	37.602	[12.44, 62.77]
High × 2014	50.974	[18.90, 83.05]	53.214	[24.55, 81.88]
Wife’s age			−6.467	[−7.21, −5.73]
Wife working (vs. not working)			−91.152	[−97.96, −85.24]
Husband weekly working hours (0–44)				
45–60			−5.878	[−12.79, 1.04]
61 or more			−4.924	[−12.99, 3.14]
Farm household (vs. not)			30.836	[12.32, 49.35]
Owning home (vs. not)			−13.803	[−19.62, −7.99]
Number of children aged 10 to 18			−32.058	[−38.88, −25.24]
Number of time diary for the weekend (ref: 0)				
1	2.189	[−4.86, 9.24]	−1.038	[−7.33, 5.25]
2	3.300	[−5.48, 12.08]	3.993	[−6.85, 8.80]
Constant	149.127	[141.50, 156.75]	193.332	[183.87, 202.80]
R^2^	0.077		0.268	
N	7,022 households			

## References

[R1] Altintas E (2016). The widening education gap in developmental child care activities in the United States, 1965–2013. Journal of Marriage and Family 78(1): 26–42. doi:10.1111/jomf.12254.

[R2] Brinton MC (ed.) (2001). Women’s working lives in East Asia. Stanford: Stanford University Press.

[R3] Dotti Sani GM and Treas J (2016). Educational gradients in parents’ child-care time across countries, 1965‒2012. Journal of Marriage and Family 78(4): 1083–1096. doi:10.1111/jomf.12305.

[R4] Gracia P (2014). Fathers’ child care involvement and children’s age in Spain: A time use study on differences by education and mothers’ employment. European Sociological Review 30(2): 137–150. doi:10.1093/esr/jcu037.

[R5] Kalil A, Ryan R, and Corey M (2012). Diverging destinies: Maternal education and the development gradient in time with children. Demography 49(3): 1361–1383. doi:10.1007/s13524-012-0129-5. 22886758 PMC4894844

[R6] Lee Y-J, Park K, and Sanida I (2020). Educational background, gender-role attitudes, and parenting time for young children. In: Park H and Woo H (eds.). Korean families yesterday and today. Ann Arbor: University of Michigan Press: 185–209.

[R7] McLanahan S (2004). Diverging destinies: How children are faring under the second demographic transition. Demography 41(4): 607–627. doi:10.1353/dem.2004.0033. 15622946

[R8] OECD (Organisation for Economic Cooperation and Development) (2019). Rejuvenating Korea: Policies for a changing society. Paris: OECD.

[R9] Park H, Byun S, and Kim K (2011). Parental involvement and students’ cognitive outcomes in Korea: Focusing on private tutoring. Sociology of Education 84(1): 3–22. doi:10.1177/0038040710392719.

[R10] Park H and Lee JK (2017). Growing educational differentials in the retreat from marriage among Korean men. Social Science Research 66: 187–200. doi:10.1016/j.ssresearch.2016.10.003. 28705355

[R11] Park H and Raymo JM (2013). Divorce in Korea: Trends and educational differentials. Journal of Marriage and Family 75(1): 110–126. doi:10.1111/j.1741-3737.2012.01024.x. 23440624 PMC3578605

[R12] Park SJ and Abelmann N (2004). Class and cosmopolitan striving: Mothers’ management of English education in South Korea. Anthropological Quarterly 77(4): 645–672. doi:10.1353/anq.2004.0063.

[R13] Shavit Y and Park H (2016). Introduction to the special issue: Education as a positional good. Research in Social Stratification and Mobility 43: 1–3. doi:10.1016/j.rssm.2016.03.003.

[R14] Song Y (2011). Changes in parental time spent with children. Korea Journal of Population Studies 34(2): 45–64 (in Korean).

[R15] Weininger EB, Lareau A, and Conley D (2015). What money can’t buy: Class resources and children’s participation in organized extracurricular activities. Social Forces 94(2): 479–503. doi:10.1093/sf/sov071.

